# Parsonage-Turner syndrome following post-exposure prophylaxis

**DOI:** 10.1186/1471-2474-15-265

**Published:** 2014-08-07

**Authors:** Duncan P Fransz, Casper P Schönhuth, Tjeerd J Postma, Barend J van Royen

**Affiliations:** 1 Department of Orthopaedic Surgery, VU University Medical Center, PO Box 7057, 1007 MB Amsterdam, The Netherlands; 2 Department of Neurology, VU University Medical Center, PO Box 7057, 1007 MB Amsterdam, The Netherlands

**Keywords:** Parsonage-Turner syndrome, Brachial plexus neuritis, Neuralgic amyotrophy, Vaccination, Scapular winging

## Abstract

**Background:**

The ‘Parsonage-Turner syndrome’ (PTS) is a rare but distinct disorder with an abrupt onset of shoulder pain, followed by weakness and atrophy of the upper extremity musculature, and a slow recovery requiring months to years. To our best knowledge, this is the first case describing symptoms and signs of PTS following the administration of a post-exposure prophylaxis (PEP) regimen against possible human immunodeficiency virus (HIV) and hepatitis B virus (HBV) infection.

**Case presentation:**

A 25-year-old Caucasian man presented with pain and unilateral scapular winging following PEP against possible HIV and HBV infection. Although atrophy and weakness were observed for the right supraspinatus muscle, a full range of motion was achievable. Neurological examination, plain radiography of the right shoulder and electromyography showed no additional abnormalities. The patient was diagnosed with post-vaccination PTS and treated non-operatively. During the following 15 months the scapular winging receded and full muscle strength was regained.

**Conclusion:**

Parsonage-Turner syndrome is a rare clinical diagnosis. The precise pathophysiological mechanism of PTS remains unclear, but it seems to involve an interaction between genetic predisposition, mechanical vulnerability and an autoimmune trigger. An immunological event, such as – in this case – a vaccination as part of PEP treatment, can trigger the onset of PTS. The clinical presentation is distinctive with acute severe pain followed by patchy paresis, atrophy and sensory symptoms that persist for months to years. No currently available tests can provide a definite confirmation or exclusion of PTS. Routine blood examination, electromyography (EMG), and computed tomography (CT) or magnetic resonance imaging (MRI) serve mainly to exclude other disorders. The recovery can be quite lengthy, non-operative treatment is the accepted practice. Supplementary administration of oral prednisolone could shorten the duration of pain. Although the outcome is typically preferable, a substantial amount of patients are left with some residual paresis and functional impairment.

## Background

The ‘Parsonage-Turner syndrome’ (PTS), ‘brachial plexus neuritis’ or ‘neuralgic amyotrophy’ is a rare but distinct peripheral nervous system disorder that may occur in otherwise healthy individuals. It is named for Maurice Parsonage and John Aldren Turner, who in 1948 reported 136 cases with a syndrome that: ‘*without any constitutional disturbance pain starts suddenly across the top of the shoulder-blade and may radiate down the outer side of the upper arm or into the neck … then a flaccid paralysis of some of the shoulder girdle … develops’*[1]. Core features include an abrupt onset of shoulder pain (usually unilaterally), followed by motor involvement (weakness and atrophy) of the upper extremity musculature, and a slow recovery requiring months to years. The extent and distribution of affected peripheral nerves can vary greatly [1-3]. In addition to the characteristic course of symptomatology, the most common tell-tale sign of the disorder is winging of the shoulder blade, which is present in approximately two-third of the patients [2]. The anterior interosseus and suprascapular motor nerves and the lateral antebrachial cutaneous and superficial radial sensory nerves are frequently involved. Sensory symptoms are usually moderate, but almost 80% of the patients can recall hypaesthesia [2,4].

Currently, no test can definitely confirm or exclude PTS. Routine blood examinations, CT or MRI, and electromyography serve mainly to exclude other disorders. Nerve conduction studies are of limited value in the diagnosis of PTS [2].

The recovery rate differs, but in general patients recover 80-90% of their full strength after 2–3 years. However, more than 70% are left with residual paresis and functional impairment. These biomechanical changes increase the risk of joint pathology and strain of the paretic and compensating muscles [2]. Although the recovery can be quite lengthy, non-operative treatment is the accepted therapy. Anecdotal evidence and a single retrospective case series show some evidence to suggest that early oral corticosteroid therapy may have a positive influence on pain in some patients, and possibly speed up recovery in a few [5]. If functional impairment persists for a prolonged period of time, surgical options have been described [6,7].

The precise pathophysiological mechanism remains unclear, but it seems to involve an interaction between an underlying genetic predisposition, a mechanical vulnerability and an autoimmune trigger (i.e. vaccination) [2,8].

Accurately diagnosing Parsonage-Turner syndrome can be difficult because of its clinical presentation. We describe a patient with PTS following the administration of post-exposure prophylaxis against possible human immunodeficiency virus and hepatitis B virus infection.

## Case presentation

**Figure 1:**
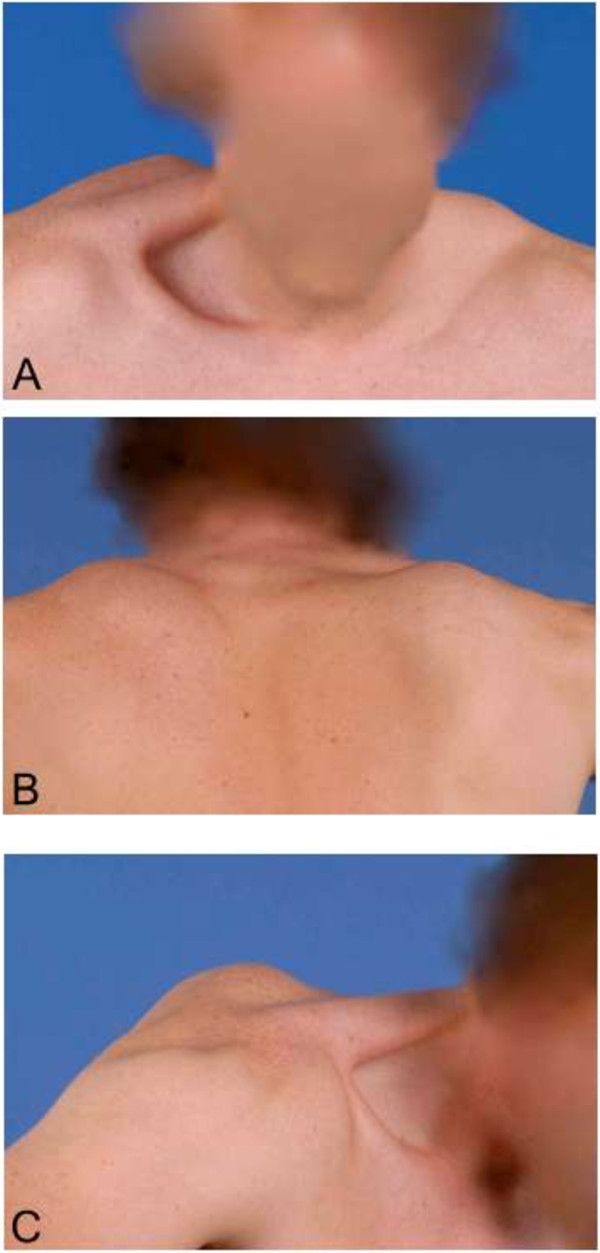
**Clinical presentation at first consultation. A**, **B** &**C**. Scapular winging in frontal, dorsal and sagittal plane. Photographs were taken at the time of presentation.

**Figure 2:**
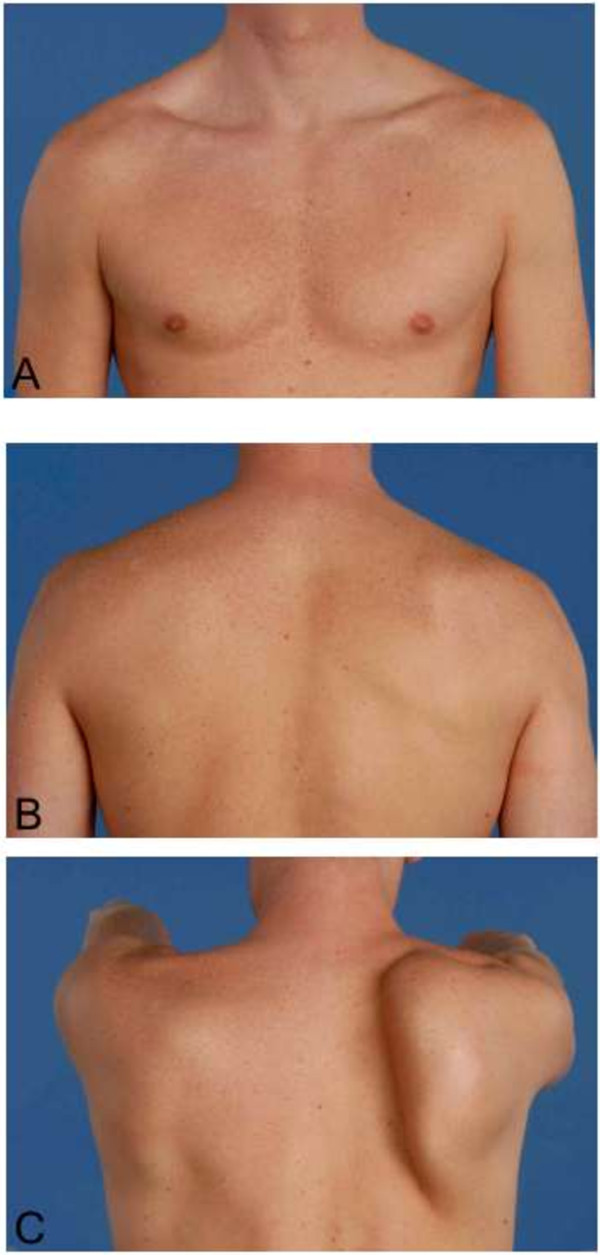
**Clinical presentation after 3 months. A**, **B** &**C**. Scapular winging and atrophy of the supraspinatus muscle. Photographs were taken three months after initial presentation.

**Figure 3:**
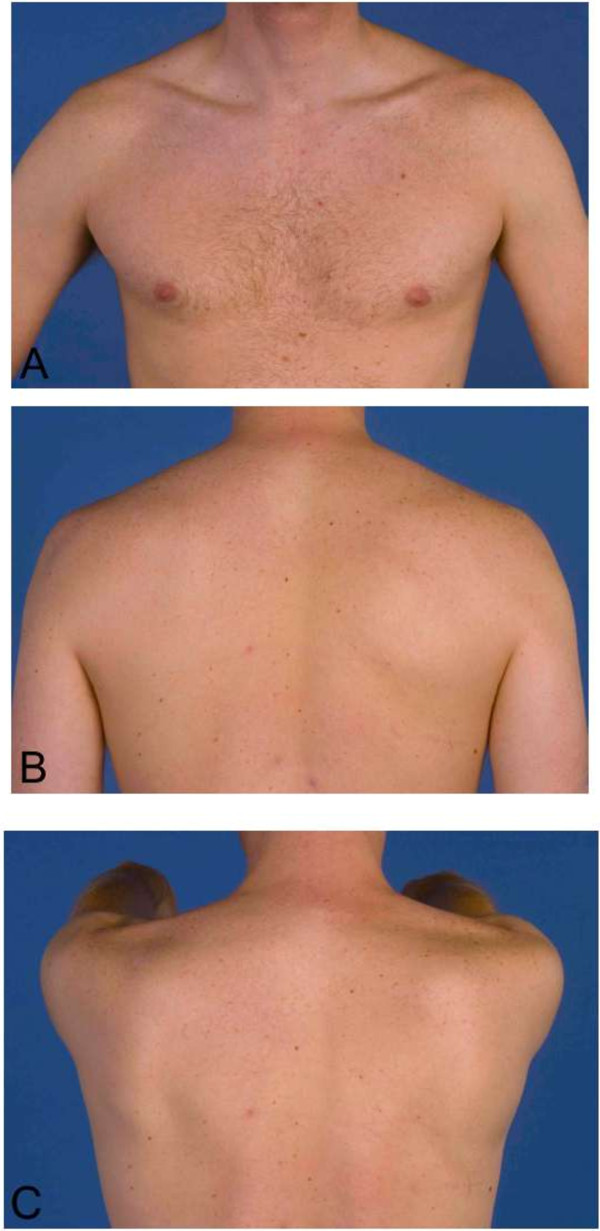
**Clinical presentation after 15 months. A**, **B** &**C**. Complete recovery of scapular winging and no residual atrophy. Photographs were taken fifteen months after initial presentation.

A 25-year-old Caucasian man was referred to our outpatient clinic, two months after initial presentation at the Emergency Room because of accidental exposure to bloodborne pathogens. In accordance with the Dutch post-exposure prophylaxis guidelines, post-exposure prophylaxis (PEP) was indicated against possible infection with human immunodeficiency virus (HIV) and hepatitis B virus (HBV) [9]. The patient immediately received 500 IU of human hepatitis B immunoglobulin intramuscularly into the right shoulder, and hepatitis B vaccination according to the vaccine dose schedule, at 0, 1 and 6 months. Additionally, the patient received 4 weeks of atazanavir 400 mg with combivir 450 mg (lamivudine/zidovudine) twice daily. Flu-like symptoms occurred in the first week, which subsequently diminished. Following the second intramuscular vaccine dose, the patient complained of neck pain, with radiating pain towards his right shoulder. The patient noticed a deviated position of his right scapula. During the next weeks the pain gradually subsided, but the scapular deviation persisted. No subjective signs of muscle weakness or sensory symptoms were present.On physical examination, the patient had evident scapular winging (Figures 1A-C). Atrophy and weakness were observed for the right supraspinatus muscle. However, a full range of motion was achievable. Thorough neurological examination did not indicate any further deficits. Plain radiography of the right shoulder showed no abnormalities. Electromyography (EMG) recorded two months after initial presentation, showed no neuromuscular abnormalities of the long thoracic or suprascapular nerve.The clinical diagnosis ‘Parsonage-Turner syndrome’ was made. The patient received non-operative treatment. The scapular winging receded during the following 15 months (Figures 2A-C and 3A-C). Full muscle strength was regained. The HIV and HBV serology proved to be negative.

## Conclusions

### Clinical presentation

Parsonage-Turner syndrome is a clinical diagnosis and presents in 96% of the cases with acute, severe neurologic pain in the upper extremities, neck and/or trunk, without any antecedent trauma. The pain usually causes the patient to wake up early in the morning, and lasts for 4 weeks on average, but disappears within 24 hours in 5% of the cases. In addition to the pain, there is a patchy distribution of motor and sensory symptoms. The most frequently involved nerves are: long thoracic nerve, anterior interosseus nerve, suprascapular nerve, lateral antebrachial cutaneous nerve and superficial radial nerve. Involvement of the long thoracic nerve leads to weakness of the serratus anterior muscle and winging of the scapula [2]. It can take quite some time to arrive at the proper diagnosis, with a median period to diagnosis of 10.5 weeks. The difficulty lies with the recognition of the clinical entity of Parsonage-Turner syndrome, especially when patients present without the indicative scapular winging [4]. Table 1 presents the differential diagnosis of PTS includes pathology of both orthopaedic and neurological nature, based on van Alfen et al. [2].

**Table 1:** **The differential diagnosis of Parsonage-Turner syndrome, based on Van Alfen et al.**[2]

***Orthopaedic disorders***	***Distinguishing features***
Rotator cuff pathology	Subacute or insidious onset, pain intensity varies, worsens with motion, weakness, progressive
Adhesive capsulitis	Subacute onset, pain, ‘frozen shoulder’, limited range of motion (active and passive), progressive
Subacromial bursitis	Subacute onset, pain along front and side, nighttime pain, painful arc of motion, fluctuating course
Calcific tendinitis	Subacute onset, pain, worsens with elevation, sometimes stiffness, self-limiting
Osteoarthritis	Insidious onset, pain, worsens with motion, stiffness, secondary weakness, slowly progressive
Facioscapulohumeral dystrophy	Onset during adolescence, facial weakness, weakening biceps/triceps/deltoids, hearing loss, painless, progressive
Cervical spondylosis with referred brachialgia	Often posture or activity dependent, no neurological deficits, fluctuating course
(Primary) tumors of the scapula	Non acute onset, scapular winging without weakness and no neurological symptoms
*Neurological disorders*	*Distinguishing features*
Cervical radiculopathy, degenerative	Insidious onset, slowly progressive or fluctuating course
Cervical radiculopathy, discrupture	Acute onset, pain varies with posture: pain, sensory and motor symptoms occur in the same dermatome
Mononeuritis multiplex/vasculitis	Symptoms also occur in legs or distal arm, subacute onset, progressive
Multifocal motor neuropathy	Painless, no sensory symptoms, distal predominance, progressive
Asian tick-borne encephalitis (poliomyelitic)	Following viral prodrome, severe headache and back pain, flaccid shoulder girdle paralysis
Focal motor neuron disease	Insidious onset, no sensory symptoms, painless, progressive
Entrapment neuropathies	Subacute onset, mild to moderate pain, prominent sensory symptoms
*Other disorders*	*Distinguishing features*
Complex regional pain syndrome	Subacute onset, vasomotor features predominate, diffuse pain and weakness, progressive
Lyme disease	Subacute onset, swelling, pain, rash, fever, fatigue, fluctuating course

### Ancillary diagnostic studies

No currently available tests can provide a definite confirmation or exclusion of PTS. Routine blood examination, electromyography (EMG), and computed tomography (CT) or magnetic resonance imaging (MRI) serve mainly to exclude other disorders such as cervical disc herniation, rotator cuff pathology or neoplasms. Both EMG and MRI must be interpreted with consideration of the clinical history.

The use of brachial plexus sensory nerve conduction studies seem to be of little diagnostic value in Parsonage-Turner syndrome. Sensory symptoms occur in 80% of the patients, sensory nerve conduction studies showed abnormalities in less than 20% of nerves, even when the nerve was clinically affected. This could be due to some sensory lesions in the nerve roots instead of the plexus. An examination of normal sensory nerve conduction studies does not rule out PTS as a diagnosis [10].

The MRI finding most typical of PTS is that of diffuse high signal intensity involving one or more muscles innervated by the brachial plexus depicted on T2-weighted images. This is thought to reflect denervation injury, with the signal intensity increase due to increased capillary blood volume in partially denervated muscle. In addition, T1-weighted images can show focal fatty atrophy in about 30% of the cases. However, both findings are not specific for PTS, but can also be caused by trauma, entrapment neuropathy and herniated cervical discs [11].

### Treatment

During the onset of symptoms, the administration of oral prednisolone could shorten the duration of pain and also accelerate the recovery in some patients. A daily dose of oral prednisolone of 1 mg/kg for one week, in combination with a long acting opioid and a non-steroidal anti-inflammatory drug (NSAID) (both twice per day) can be considered [5]. The musculoskeletal pain that ensues from altered biomechanics of the affected extremity can be treated by a combination of physiotherapy for posture, mobility and relaxation, and NSAIDs. The patient should be encouraged to use the affected extremity as fully as possible [2,5].

Although recovery can be quite lengthy, non-operative treatment is the accepted practice. The rare patient who doesn’t improve, may need surgical intervention [3,12]. Decompression and microneurolysis of the long thoracic nerve in the supraclavicular spaces has shown improvement with regard to scapular winging, pain reduction and shoulder instability [13]. Another accepted surgical procedure for non-resolving scapular winging is a dynamic muscle transfer in which the sternal head of the pectoralis major is transferred to the inferior angle of the scapula extended or reinforced by a fascial autograft. This procedure has shown consistently positive results with improved function, resolution of winging, and relief of pain [6,7].

### Outcome

The severity of onset, development and extent of recovery can vary between patients. Overall, most patients recover 80-90% of their full strength after 2–3 years, but more than 70% are left with some residual paresis and functional impairment. These biomechanical changes possibly increase the risk of joint pathology and strain of the paretic and compensating muscles [2]. Almost a third of the patients suffer from chronic pain, and the majority exhibits persisting functional deficits after an average follow-up of more than six years. It is therefore recommended that the attending physician communicates this possibility with the patient at an early stage [4].

### Pathophysiology

The pathophysiological mechanisms seem to involve an interaction between an underlying genetic predisposition, a mechanical vulnerability, and an autoimmune trigger. The direct relationship between the genetic predisposition and the actual onset of PTS is still unknown [2]. Nearly 10% of the manifestations of PTS are preceded by unusual physical exercise. This role for mechanical factors was demonstrated during an epidemic of PTS, which occurred when a contaminated water supply infected hundreds of people. However, PTS mainly occurred among workers in a nearby knitting factory, who used their arms strenuously [14]. The onset of PTS could be mediated by wear-and-tear-induced weakening of the blood-nerve barrier that normally prevents any contact with the peripheral nervous system [2]. In more than 50% of the patients with PTS different types of immunological events, such as vaccination, surgery, pregnancy, childbirth, immunotherapy and many different types of infection, have been reported [4].

With regard to the aforementioned patient, it is most likely that the post-exposure prophylaxis against HIV and HBV triggered the onset of PTS, as the serology proved to be negative for infection.

There is limited literature available concerning PTS and HIV. One case report described the occurrence of PTS as a rare hypersensitivity reaction of HIV-infected patients to abacavir [15], a drug our patient did not receive. Two case reports describe the onset of PTS in the context of acute HIV seroconversion illness [16,17]. Another study reports a case of varicella reactivation causing bilateral PTS in an HIV-positive patient [18].

With regard to PTS and HBV, a three-year postmarketing surveillance for neurologic adverse events following plasma derived hepatitis B vaccination (each 1.0 ml of vaccine contained 20 μg of hepatitis-B surface antigen (HBsAg)) was carried out in 1988 for 850.000 patients. A total of 41 neurologic adverse events were reported: Bell's palsy (10), Guillain-Barré syndrome (9), convulsions (5), lumbar radiculopathy (5), optic neuritis (5), transverse myelitis (4), and Parsonage-Turner syndrome (3). Half of these events occurred after the first of three required vaccine doses [19]. In addition, there is one case report that describes the occurrence of PTS following recombinant DNA (deoxyribonucleic acid) hepatitis B vaccination (which consisted of 20 μg of the purified HBsAg) [20].

There are also reports that describe PTS following various other vaccinations, such as influenza [21,22], human papilloma virus [23,24], DPT (diphtheria, pertussis, tetanus) [22,25,26], swine flu [27] and tetanus [22,28]. Similarly, the occurrence of PTS following an infection has been described on multiple occasions. Table 2 presents an overview of the micro-organisms that have been associated with PTS, based on Stek et al. [29] and augmented with recent studies [29,30].

**Table 2:** **The micro-organisms associated with Parsonage-Turner syndrome, based on Stek et al.**[29]

***Viruses* Herpes simplex**	***Bacteria*** **Escherichia coli**	***Moulds*** **Aspergillus species**
Epstein-Barr	Borrelia burgdorferi	
Cytomegalo	Neisseria gonorrhoe	
Varicella zoster	Salmonella panama	
Parvo B19	Yersinia enterocolica	
Human immunodefiency	Staphylococcus aureus	
Hepatitis B	Streptococcus group A	
Hepatitis E	Brucella species	
Vaccinia	Coxiella burnetti	
Coxsackie B	Chlamydophila pneumoniae	
West Nile	Leptospira species	
Dengue fever	Mycoplasma pneumoniae	
	Bartonella henselae	

To our knowledge, this is the first case report describing the occurrence of Parsonage-Turner syndrome following the administration of post-exposure prophylaxis against possible infection with HIV and hepatitis B. Similarly to the cases previously described [19,20], the HBV vaccination most likely caused PTS in our patient. Non-operative treatment is the standard treatment, and although the outcome is typically preferable, almost a third of the patients suffer from residual complaints after six years.

### Patient consent

“Written informed consent was obtained from the patient for publication of this Case report and any accompanying images. A copy of the written consent is available for review by the Editor of this journal”.

## Abbreviations

PTS: Parsonage-Turner syndrome; PEP: Post-exposure prophylaxis; HIV: Human immunodeficiency virus; HBV: Hepatitis B virus; EMG: Electromyography; CT: Computed tomography; MRI: Magnetic resonance imaging; NSAID: Non-steroidal anti-inflammatory drug; HBsAg: Hepatitis-B surface antigen; DNA: Deoxyribonucleic acid; DPT: Diphtheria, pertussis, tetanus.

## Competing interests

The authors declare that they have no conflict of interest and sources of financial support to the publication of this article.

## Author’s contributions

DPF: Conception and design, analysis and interpretation, writing the manuscript. CPS: Conception and design, analysis and interpretation, critical revision of the manuscript, supervision. TJP: Data collection, analysis and interpretation, critical revision of the manuscript. BJR: Conception and design, data collection, critical revision of the manuscript, supervision. All authors read and approved the final manuscript.

## Pre-publication history

The pre-publication history for this paper can be accessed here:

http://www.biomedcentral.com/1471-2474/15/265/prepub
